# Standardized patients in psychiatry – the best way to learn clinical skills?

**DOI:** 10.1186/s12909-018-1184-4

**Published:** 2018-04-06

**Authors:** Monika Himmelbauer, Tamara Seitz, Charles Seidman, Henriette Löffler-Stastka

**Affiliations:** 1 Teaching Center, Medical University of Vienna, Vienna, Austria; 2 Department of Infectious Diseases and Tropical Medicine, SMZ Süd, Kundratstraße 10, 1100 Wien, Austria; 3 Columbia University, New York, USA; 4 Department of Psychoanalysis and Psychotherapy, and Teaching Center/Postgraduate Unit, Medical University of Vienna, Währinger Gürtel 18-20, 1090 Vienna, Austria

**Keywords:** Standardized Patients, SP, Simulated patient, Communication skills, Medical teaching, Doctor-patient-talk, Psychiatry, Taking medical history

## Abstract

**Background:**

Standardized patients (SP) have been successfully utilized in medical education to train students’ communication skills. At the Medical University of Vienna communication training with SPs in psychiatry is a mandatory part of the curriculum. In the training, the SP plays the role of four different patients suffering from depression/suicidal tendencies, somatoform disorder, anxiety disorder, or borderline disorder while the student attempts to gather the patient’s medical history. Both the instructor and SP then give the student constructive feedback afterwards.

**Method:**

The aim of the study was to evaluate the quality of the SP’s roleplay and feedback, using a self-created questionnaire. Additionally, we wanted to gauge the differences between the students’ and teachers’ evaluations of the SP’s role playing performance and feedback.

**Results:**

The questionnaire was completed by 529 students and 29 teachers who attended the training. Overall, both students and teachers evaluated the SPs’ performance and feedback very well. In comparison to the responses given by the teachers, more students reported that the “SP overacted” while fewer students believed that the “SP could be a real patient”. The feedback given by the SP was evaluated similarly by students and teachers, suggesting that students are able to recognize the quality of constructive feedback. Furthermore, the SP’s quality of roleplaying was evaluated as the poorest while playing the psychiatric disorder “depression/suicidal tendencies.”

**Conclusions:**

Our study showed that students and teachers appreciate SPs’ competence of role play and of giving feedback. However, further studies should be performed to figure out why both students and teachers alike evaluated the played psychiatric disorder “depression/suicidal tendencies” to be the worst.

## Background

Standardized patients (SPs) have been used in medical education for almost 50 years [1]. Previous literature has demonstrated that training with SPs in medical education is a valuable tool [2], correlating with a high learners’ satisfaction [3] and an improvement in learners’ understanding of certain topics and skills [4]. Training with SPs prepared students to handle a broad range of psychiatric patients, especially those who suffer from alcohol abuse [5], suicidal tendencies [6], and patients at the end of their lives [7]. Additionally, SP training is indispensable in preparing students to effectively communicate bad news to patients [8].

Several medical universities offer training with SPs [3]. At the Medical University of Vienna, the implementation of communication training with SPs in psychiatry commenced in 2012/2013. At this University, standardized patients are professional actors who are trained to act as patients with specific medical conditions, as well as a defined medical and life history. They have regular mandatory trainings for learning new roles, refreshing established roles, and practising the giving of feedback. In addition, there are specific preparation courses for their appearance in lessons and exams. Furthermore, for standardizing SPs’ acting, communication, and feedback skills, an appropriately skilled instructor is responsible [9]. In each training session, the SP plays different clinical scenarios, each of them suffer from one of the following diseases:

1. Depression/suicidal tendencies
2. Somatoform disorder
3. Anxiety disorder
4. Borderline disorder

The student’s aim is to take a basic medical history, while tailoring their questions in accordance with the patient’s disease. The challenge for the SP is in both playing the patient authentically and giving the students constructive feedback afterwards [10]. As a function of the case and the individual communication between student and SP (role play and feedback) there are 4 to 6 student-SP interactions per lesson. Quality assurance and supervision during regular mandatory trainings were conducted by the peer actors and the SP trainer (who is a specially trained actor). It contains observation and monitoring of SP’s roleplay as well as feedback for SP.

The courses with standardized patients take place in small groups. All groups consist of about 20 students, a course instructor and a standardized patient. The lesson proceeds as follows: During the class, every student has several conversations with the standardized patient. After the conversation, the focus is on constructive feedback. First, the student reflects on the conversation himself/herself; second, the standardized patient, fellow students, and course instructor provide the student with feedback [9].

The students have the opportunity to prepare themselves for the individual diseases by means of a textbook [11]. With the help of the clinical scenarios, which are played by the SP in the course, the students can focus on the problem areas (e.g., recognizing suicidal tendencies, recognizing drug dependencies) for each particular disorder. Each case presentation is broken down into specific areas that the students must record: symptom characteristics, life circumstances, personality traits and social situation, medical history and current situation. Further information on the theoretical background of the individual clinical scenarios (epidemiology, genetics, clinics, psychopathology, neurophysiology, pathophysiology, psychopharmacology and psychotherapy) are given [11]. Also e-ressources [12] (e.g. training e-cases, tutorials, instructive movies) are offered. But concrete prior experiences with real patients or patient presentations are not given.

The major goal of the Standardized Patients (SP) Program at the Medical University of Vienna is to effectively train the SP actors to play the role of a patient and to give the students beneficial feedback. An optimal and authentic roleplay as well as adequate feedback facilitates an increase in the students’ communication skills, especially in the field of psychiatric exploration [13].

The primary aim of this study is to evaluate the quality of SP’s roleplaying through analysis of the feedback given by the students and teachers who attended the training. The secondary aim is to assess the differences between the evaluations regarding the played psychiatric disease. For example, might such a complex disease like the borderline personality disorder be more difficult to play than the others, leading to a less convincing performance? Some of our SP’s told us that the borderline patient is hard to play, making it more difficult for them to give the student adequate feedback, concerning how he or she (the student) could better approach a patient with this disorder. Solving this problem could be a challenge for prospective actors and for our SP trainers. The final aim is to evaluate differences in the assessment of SPs’ roleplay and feedback between students and teachers. We assumed that teachers as psychiatrists or clinical psychologists have more clinical experience, meaning they can estimate the authenticity of the actors’ role play better and more consistently than students. However, we assumed that there is no discrepancy between students’ and teachers’ ability to rate the constructive feedback given by the SP, because both students and teachers understand the feedback rules.

## Methods

### Study design

A questionnaire to evaluate the quality of the SP’s role play and the feedback was developed. The questionnaire was partly based on two existing questionnaires used in the Netherlands, the Nijmengen Evaluation of Simulated Patient [14] and Maastricht Assessment of Simulated Patient [15]. The questionnaire used in this study (see Table 1) consisted of 14 statements, 7 about the quality of the SP’s roleplaying and 7 about the quality of their feedback. Excluded from the Nijmengen and Maastricht inventories were items, which address the SP as an instructor. At the Medical University of Vienna the teachers are the instructors, while the SPs are only required to play the role and give feedback.

**Table 1:** Questionnaire

Roleplaying
The SP played his/her role authentically.
The SP overacted.
The SP could be a real patient.
The SP stayed in his/her role the whole time.
The SP challenged the student.
The SP adjusted role on student’s level.
The SP’s appearance fitted to the played role.
Feedback
The SP gave feedback from the patient’s point of view.
The SP gave feedback regarding the student’s behaviour.
The SP gave constructive criticism and suggestions for improvement.
The SP gave examples about good and bad aspects during the conversation.
The SP said how he/she felt during the conversation.
The SP communicated with “I-messages”
The SP behaved respectfully towards the student.

Assessment of the SP’s quality of roleplaying and feedback

Statements were graded on a 4-point scale, with 1 representing “I agree very much”, and 4 representing “I do not agree at all”. An additional question allowed the participants to give an overall assessment of the SP. This question was rated on a 5-point scale of 1 to 5, with 1 being “very good” and 5 being “very bad”. The standardized Cronbach’s alpha was .713 for the role playing scale and .706 for the feedback scale.

### Study sample

A total of 601 students in the fourth academic year at the Medical University of Vienna und 32 teachers were asked to fill out the questionnaire after completing the “Doctor-patient-communication in psychiatric settings” workshop-seminars. There were 32 groups in total, 1 teacher per 20 students, and 1 SP. 21 SPs were evaluated.

### Statistics

Demographic data of students, teachers, and SPs were collected. Due to the small number of teachers, a complex variance analysis was performed for just the group of students. For students, a “mixed model” was developed with the rated mean score of role play and feedback as the dependent variable and the psychiatric disorder as the independent variable. A 5% level of significance was used for all tests and the analyses were performed using SAS 9.4, R3.1.3 and SPSS 21.0.

## Results

### Characteristics of the study population

The questionnaire was completed by 529 students and 29 teachers, resulting in a response rate of 88% and 91% respectively. The median age of the students was 23 years, 55% of whom were female and 45% of whom were male. In the group of teachers, whose ages ranged from 26 to 64 years, 79% were female and 21% were male. Of the actors, ages between 30 and 75, 68% were female and 32% were male. The number of the observed disorders was balanced, with each disorder being acted out an equal number of times.

### Evaluation of quality of SP’s roleplaying

Overall, students and teachers evaluated the quality of the SP’s role playing very well. Only the statements “SP challenged the students” and “SP adjusted role on students’ level” were rated as “rather good” by both. Tables 2 and 3 show the measures of central tendency and of distribution.

**Table 2:** Evaluation of quality of SP’s roleplaying (students). (Scale: 1 = “very good” to 5 = “very bad”)

Items	N	NMISS	Min	Q1	Median	Q3	Max
The SP played his/her role authentically.	527	2	1	1	1	1	4
The SP overacted. (recoded)	527	2	1	1	2	2	4
The SP could be a real patient..	525	4	1	1	2	2	4
The SP stayed in his/her role the whole time.	529	0	1	1	1	1	4
The SP challenged the student.	523	6	1	2	2	2	4
The SP adjusted role on student’s level.	523	6	1	2	2	2	4
The SP’s appearance fitted to the played role.	525	4	1	1	1	2	4

**Table 3:** Evaluation of quality of SP’s roleplaying (teachers). (Scale: 1 = “very good” to 5 = “very bad”)

Items	N	NMISS	Min	Q1	Median	Q3	Max
The SP played his/her role authentically.	29	0	1	1	1	2	2
The SP overacted. (recoded)	29	0	1	1	1	2	4
The SP could be a real patient..	29	0	1	1	1	2	3
The SP stayed in his/her role the whole time.	29	0	1	1	1	1	2
The SP challenged the student.	29	0	1	1	2	2	3
The SP adjusted role on student’s level.	29	0	1	1	2	2	4
The SP’s appearance fitted to the played role.	29	0	1	1	1	2	3

### Differences between students’ and teachers’ evaluations

Only a slight difference between students’ and teachers’ evaluations was observed (Cohen’s d = 0.27). The only significant difference appeared in the statements “SP overacted”, to which more students agreed, and “SP could be a real patient”, to which more students disagreed.

### Differences between the students’ evaluations regarding the played psychiatric disease

**Fig. 1:**
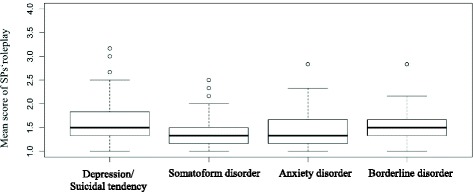
Boxplots for the mean score of roleplay per clinical picture (students)

Pair-by-pair comparisons showed a significant difference between the evaluation of the played psychiatric disease “anxiety disorder” and “depression/suicidal tendencies” (adjusted *p* = .0096, Cohen’s d = .202), as well as between “depression/ suicidal tendencies” and “somatoform disorder” (adjusted *p* = .012, Cohen’s d = .225). The SP’s performance when playing the psychiatric disorder “depression/suicidal tendencies” was evaluated significantly to be worse than their portrayal of both “anxiety disorder” or “somatoform disorder”. All other combinations of disorders showed no significant difference in evaluation. In Fig. 1 boxplots for the mean score of roleplay per clinical scenario are shown. However, the effects were small: The adjusted 95% confidence interval of depression/suicidal tendencies vs. anxiety disorder was -0.20 [-0.37;-0.036] and of depression/suicidal tendencies vs. somatoform disorder 0.17 [0.027;0.31].

### Evaluation of the quality of the SP’s feedback

Both, students and teachers evaluated the quality of the actor’s feedback very well. Only the statement “SP gave constructive criticism and suggestions for improvement” was rated as rather good by both. No significant difference could be shown between the students’ and teachers’ evaluations of the SP’s feedback. Tables 4 and 5 show the measures of central tendency and of distribution.

**Table 4:** Evaluation of quality of SP’s feedback (students). (Scale: 1 = “very good” to 5 = “very bad”)

Items	N	NMISS	Min	Q1	Median	Q3	Max
The SP gave feedback from the patient’s point of view.	525	4	1	1	1	1	4
The SP gave feedback regarding the student’s behaviour.	523	6	1	1	1	2	4
The SP gave constructive criticism and suggestions for improvement.	524	5	1	1	2	2	4
The SP gave examples about good and bad aspects during the conversation.	524	5	1	1	1	2	4
The SP said how he/she felt during the conversation.	525	4	1	1	1	2	4
The SP communicated with “I-messages”.	517	12	1	1	1	2	4
The SP behaved respectfully towards the student.	525	4	1	1	1	1	4

**Table 5:** Evaluation of quality of SP’s feedback (teachers). (Scale: 1 = “very good” to 5 = “very bad”)

Items	N	NMISS	Min	Q1	Median	Q3	Max
The SP gave feedback from the patient’s point of view.	29	0	1	1	1	2	2
The SP gave feedback regarding the student’s behaviour.	29	0	1	1	1	2	2
The SP gave constructive criticism and suggestions for improvement.	29	0	1	1	2	2	3
The SP gave examples about good and bad aspects during the conversation.	29	0	1	1	2	2	3
The SP said how he/she felt during the conversation.	29	0	1	1	1	2	3
The SP communicated with “I-messages”.	29	0	1	1	1	2	3
The SP behaved respectfully towards the student.	29	0	1	1	1	1	4

### Differences between the students’ evaluations regarding the played psychiatric disease and the respective feedback

**Fig. 2:**
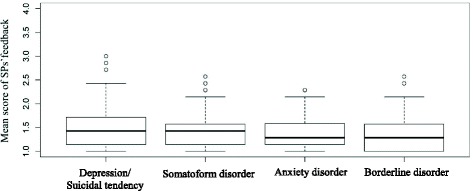
Boxplots for the mean score of feedback per clinical picture (students)

No difference between the students’ evaluations regarding the played psychiatric disease could be found (*p* = .10). In Fig. 2, boxplots for the mean score of feedback per clinical scenario are shown.

### Evaluation of SP’s overall performance

The overall assessments of the SPs were very good (Median: 1, Mean: 1.47, Standard Deviation: 0.68).

## Discussion

The data showed that the SPs’ performance in the communication training was evaluated very well by both students and teachers who attended the workshop.

The only difference between the students’ and teachers’ evaluations was that more students stated that the SP overacted and did not behave like a real patient. This discrepancy is a testament to the students’ lack of clinical experience. We assume that experienced teachers, after the training, would explain that a real patient with the specific disorder would behave very similarly to the SP. Another explanation might be that the student’s first contact with psychiatry requires a confrontation with their own feelings and behaviour [16]. This might cause the student to become afraid and exhibit a defensive attitude, leading to the student’s judgment that the SP or even a real patient exaggerated [16]. This topic should also be discussed during feedback, concentrating on the student’s reflective process [13].

The quality of the feedback given by the SP was evaluated similarly by both students and teachers, suggesting that students are able to recognize the quality of constructive feedback equally as well as teachers.

The data also indicated that the SP’s performance of a patient with depression/suicidal tendencies was evaluated to be worse than the performances of the other diseases.

There are many reasons for this. Because typical symptoms of depression are apathy and emotional withdrawal, students might evaluate the performance poorly because of a lack of understanding and empathy towards the depressive patient. Even experienced therapists have been known to devalue depressive patients in their countertransference [17]. We suggest bringing the topic of countertransference into the communication training, helping students better deal with and understand their feelings towards the patient.

Another reason for the poor evaluation could be that the quality of the role script or the guidance of roleplaying between the clinical scenarios is different.

Furthermore, the mixed design model for comparing the assessment of the four clinical scenarios was not calculated for teachers because of sample size. It is possible that the teachers would evaluate the SPs’ competence of playing the disorder depression/suicidal tendencies comparatively better than the students did.

### Limitations

A limitation of this study is the small number of SPs and teachers in each group, leading to limited validity of some of the data. Moreover, the gender distribution in the group of teachers and SPs is quite unequal.

## Conclusions

Our study showed that both students and teachers were highly satisfied with working with SPs in psychiatric settings.

Despite that, the SP’s performance as a patient with depression/suicidal tendencies was evaluated to be worse than their performance of the other diseases. We suggest, considering that depression is a very common disorder, examining the played disorder depression/suicidal tendencies more precisely.

First, we suggest collecting SP evaluation data from a larger sample of teachers. The question could be, whether teachers, in comparison to experienced psychiatrists, evaluate the SP’s performance of this specific disorder worse than that of others. Second, we suggest interviewing the SPs about how they feel playing a depressive patient and ask them to evaluate themselves. Third, students should be interviewed. They could be asked about their general feelings towards working with a depressive patient and about why they poorly evaluated the SP’s performance of the disorder depression/suicidal tendencies.

A comparison using real depressive patients could also be undertaken. Therefore, further research is needed to get insight in the students’ learning outcome.

Additionally, analysing the role play training of the four psychiatric disorders as well as the corresponding role scripts would also be reasonable. Another way to improve the training is to use Stanislawski’s Method [13], which requires the actors to be in close contact with real patients, studying their psychopathology and understanding their specific biography and pathoplastic moments.

The students’ lack of clinical experience could be supplemented by appropriate video clips of real patients and a corresponding discussion forum.

The findings of further studies should help highlight the conditions of how to best carry out communication training with SPs in psychiatry. Therefore, it is necessary that evaluations of SPs’ roleplay of clinical scenarios are conducted at other locations, because there is very little literature in this particular area.

## References

[1] Levine AI, Swartz MH (2008). Standardized patients: the “other” simulation. J Crit Care.

[2] May W, Park JH, Lee JP (2009). A ten-year review of the literature on the use of standardized patients in teaching and learning: 1996-2005. Med Teach.

[3] McNaughton N, Ravitz P, Wadell A, Hodges BD (2008). Psychiatric education and simulation: a review of the literature. Can J Psychiatry Rev Can Psychiatr.

[4] Xie H, Liu L, Wang J, Joon KE, Parasuram R, Gunasekaran J (2015). The effectiveness of using non-traditional teaching methods to prepare student health care professionals for the delivery of mental state examination: a systematic review. JBI Database Syst Rev Implement Rep.

[5] Hayes-Roth B, Saker R, Amano K (2010). Automating individualized coaching and authentic role-play practice for brief intervention training. Methods Inf Med.

[6] Fallucco EM, Hanson MD, Glowinski AL (2010). Teaching pediatric residents to assess adolescent suicide risk with a standardized patient module. Pediatrics.

[7] Szmuilowicz E, el-Jawahri A, Chiappetta L, Kamdar M, Block S (2010). Improving residents’ end-of-life communication skills with a short retreat: a randomized controlled trial. J Palliat Med.

[8] Simpson JS (2014). The educational utility of simulations in teaching history and physical examination skills in diagnosing breast cancer: a review of the literature. J Breast Cancer.

[9] http://teachingcenter.meduniwien.ac.at/abteilungen/methodik-und-entwicklung/schauspielpatientinnen-programm/. (retrieved 27.06.2017, 11:50).

[10] Bokken L, Linssen T, Scherpbier A, van der Vleuten C, Rethans J-J (2009). Feedback by simulated patients in undergraduate medical education: a systematic review of the literature. Med Educ.

[11] Löffler-Stastka H, Doering S (2015). Psychische Funktionen in Gesundheit und Krankheit. Ärztliche Gesprächsführung [Psychic functions in health and disease, doctor-patient communication] (11., überarb. und erg. Aufl.).

[12] Turk BR, Krexner R, Otto F, Wrba T, Löffler-Stastka H. Not the ghost in the machine: transforming patient data into e-learning cases within a case-based blended learning framework for medical education. Procedia - Soc Behav Sci. 2015;186:713–25. http://www.sciencedirect.com/science/article/pii/S1877042815023666. Retrieved 01.04.2018.

[13] Löffler-Stastka H, Datz F, Parth K, Preusche I, Bukowksi X, Seidman C (2017). Empathy in psychoanalysis and medical education – what can we learn from each other?. BCM Med Educ.

[14] Bouter S, van Weel-Baumgarten E, Bolhuis S (2013). Construction and validation of the Nijmegen evaluation of the simulated patient (NESP): assessing simulated patients’ ability to role-play and provide feedback to students. Acad Med J Assoc Am Med Coll.

[15] Wind LA, Van Dalen J, Muijtjens AMM, Rethans J-J (2004). Assessing simulated patients in an educational setting: the MaSP (Maastricht assessment of simulated patients). Med Educ.

[16] Löffler-Stastka H, Blüml V, Ponocny-Seliger E, Hodal M, Jandl-Jager E, Springer-Kremser M (2010). Students ’ Attitudes Towards Psychotherapy: Changes After a Course on Psychic Functions in Health and Illness [Einstellungen von Medizinstudenten zu Psychotherapie: Veränderungen nach Unterricht über Psychische Funktionen in Gesundheit und Krankheit]. Psychother Psychosom Med Psychol.

[17] Datz F, Parth K, Rohm C, Madanoglu S, Seidman C, Löffler-Stastka H (2016). Dimensions of activity in countertransference and therapist reaction: therapist reactions during sessions with depressed patients. [Reaktionsformen der Beziehungsgestaltung in der Behandlung depressiver Patienten - Gegenübertragung und therapeutische Aktivität]. Z Psychosom Med Psychother.

